# Is the glass half full or half empty? A qualitative exploration on treatment practices and perceived barriers to biomedical care for patients with nodding syndrome in post-conflict northern Uganda

**DOI:** 10.1186/s13104-015-1323-5

**Published:** 2015-08-29

**Authors:** Amos Deogratius Mwaka, Elialilia S. Okello, Catherine Abbo, Francis Okot Odwong, Willy Olango, John Wilson Etolu, Rachel Oriyabuzu, David Kitara Lagoro, Byamah Brian Mutamba, Richard Idro, Bernard Toliva Opar, Jane Ruth Aceng, Assuman Lukwago, Stella Neema

**Affiliations:** Department of Medicine, Mulago Hospital and the School of Medicine, College of Health Sciences, Makerere University, P.O Box 7072, Kampala, Uganda; Department of Psychiatry, Mulago Hospital and the School of Medicine, College of Healthcare Sciences, Makerere University, Kampala, Uganda; Global Friends, Kampala, Uganda; Soroti Regional Referral Hospital, Soroti, Uganda; School of Medicine, Gulu University, Gulu, Uganda; Butabika National Mental Referral Hospital, Kampala, Uganda; Department of Paediatrics and Child Health, Mulago Hospital and the School of Medicine, College of Health Sciences, Makerere University, Kampala, Uganda; Centre for Tropical Medicine and Global Health, University of Oxford, Oxford, UK; Ministry of Health Headquarters, Kampala, Uganda; Department of Sociology and Anthropology, Makerere University, Kampala, Uganda

**Keywords:** Nodding syndrome, Medical syncretism, Help-seeking, Barriers to health seeking, Civil conflict

## Abstract

**Background:**

Nodding syndrome has increasingly become an issue of public health concern internationally. The etiology of the disorder is still unknown and there are yet no curative treatments. We explored perceptions about treatment practices and barriers to health seeking for nodding syndrome in Pader and Kitgum districts in northern Uganda in order to provide data necessary for informing policy on treatment adherence and rehabilitations.

**Methods:**

We used focus group discussions and individual interviews to gain deep insights into help-seeking and treatment practices for nodding syndrome. Purposive sampling was used to identify information-rich participants that included village health teams, community members not directly affected with nodding syndrome, district leaders, healthcare professionals, and caregivers of children affected with nodding syndrome. We used qualitative content analysis to analyze data and presented findings under distinct categories and themes.

**Results:**

Caregivers and communities sought care from multiple sources including biomedical facilities, traditional healers, traditional rituals from shrines, and spiritual healing. Nodding syndrome affected children reportedly have showed no enduring improvement with traditional medicines, traditional rituals, and prayers. A substantial minority of participants reported minimal improvements in symptoms of convulsions with use of western medicines. Challenges involved in health seeking included; (1) health system factors e.g. long distances to facilities, frequent unavailability of medicines, few healthcare providers, and long waiting times; (2) contextual and societal challenges e.g. lack of money for transport and medical bills, overburdening nature of the illness that does not allow time for other activities, and practical difficulties involved in transporting the physically deformed and mentally retarded children to the health facilities.

**Conclusions:**

Help-seeking for nodding syndrome is pluralistic and include use of traditional and biomedical practices. Western medicines admittedly showed at least short term control on nodding syndrome symptoms, especially convulsions and led in a few cases to regain of functional abilities. However, multiple barriers hinder health seeking and interfere with adherence to biomedical treatments. Regarding cure, there are hitherto no treatments participants perceive cure nodding syndrome.

## Background

Nodding syndrome is a disorder of unknown etiology that has been described in sub Saharan African countries including South Sudan, Uganda and Tanzania [1–3]. The disorder is characterized by episodic head nodding and mainly affects children aged between 3 and 18 years [2, 4]. In Northern Uganda, the estimated total number of affected children was about 3541 in the three districts of Kitgum, Pader and Lamwo. In a population-based study in which case identification was informed by the WHO case diagnostic criteria for nodding syndrome, it was found that about 7 out of every 1000 children in Kitgum and Pader districts had nodding syndrome during 2012 and 2013 [5, 6].

Nodding syndrome has increasingly become an issue of public health concern mainly because of the chronic and progressive nature of the disease, lack of curative treatment and the physical and mental debilitations that accompany the disorder [7]. There is a conspiracy theory about etiology of nodding syndrome. The majority of participants from the affected regions believe that nodding syndrome could be a politically engineered disorder and this has led to apathy, helplessness and belief that cause and curative treatments are unknown because of purposeful neglect of health needs and lack of will to reduce suffering of people in the affected region by Uganda government [8–11].

Since the recognition of nodding syndrome in Tanzania in the early 1960s [2], in South Sudan about 2001 [1, 12] and in Uganda between 2003 and 2009 [13], there has been no curative treatment for the disorder. Community members have thus adopted several treatment practices, mostly informed by their perceived causes of the disorder. These have included use of both traditional remedies and biomedical treatments. The use of traditional and spiritual remedies may arise from perceptions that nodding syndrome is related to and probably a direct result of war, bomb fumes and associated socio-cultural disruptions [8, 9]. On the other hand, most biomedical treatments have focused on control of symptoms of head nodding and seizures with anticonvulsants including carbamazepine, phenytoin, phenobarbital, and sodium valproate [3, 14, 15]. In Uganda, specific treatment centers have been established by government for management of children with nodding syndrome in the three districts of Kitgum, Pader and Lamwo. In these centers, anticonvulsants are provided alongside nutritional supplementations and management of injuries secondary to falls. A treatment protocol was developed to guide primary healthcare professionals in treatment and monitoring outcomes and side effects [14]. Primary healthcare professionals were trained in the use of this protocol for treatment of nodding syndrome [16]. In spite of government efforts to curb nodding syndrome through training healthcare professionals and establishing treatment centers for management of nodding syndrome, access to treatment has remained a challenge and understanding details of the barriers to health seeking from perspectives of the community, local leaders and frontline healthcare professionals is crucial to informing interventions to improve access and adherence to treatment. In addition, there is limited data on community experiences on health seeking challenges by caregivers of children with nodding syndrome.

In this study we explored perceptions of the community, caregivers of children with nodding syndrome, village health teams, primary healthcare professionals and key district leaders on treatment practices and challenges faced in seeking biomedical care for nodding syndrome in two of the most affected districts in northern Uganda. This information is intended to guide the Ministry of Health in planning and improving care based on identified barriers at the operational and community levels.

## Methods

### Design and setting

This study was part of a cross sectional qualitative study conducted in northern Uganda during November and December 2013 to explore perceptions and beliefs on nodding syndrome, health seeking, and stigma associated with nodding syndrome and coping strategies.

The population in northern Uganda experienced a protracted civil conflict that lasted more than two decades. For about a decade, about 90 % of the population in northern Uganda lived in internally displaced people’s camps (IDPs) and relied on relief food and households items distributed by World Food Program (WFP) and other humanitarian organizations [17–19]. People in camps reported challenges including lack of adequate food, lack of access to healthcare resources and facilities, and poverty that affected their lives and limited their capabilities [20]. Since 2006, people have left the IDP camps and returned to their homesteads [21].

### Participants and sampling

Participants for this study were selected from two sub counties; Atanga in Pader and Akwang in Kitgum districts. The two districts and sub counties were purposefully selected based on evidence of high prevalence and first notification of nodding syndrome [22, 23]. The official notification and recognition of nodding syndrome as a public health disorder occurred in 2009 following the Uganda government ministry of health and WHO collaborative investigations into the disorder. However there are reports to the effect that the first cases of nodding syndrome were reported in Kitgum district in about 1997 [6, 24]. Therefore, communities in these districts have had long term experience of nodding syndrome and could have formed some belief patterns about nodding syndrome, adopted some treatment practices and hold shared experiences of challenges faced during health seeking for nodding syndrome.

Participants included five groups; district officials involved in planning and response to nodding syndrome, healthcare professionals working in the nodding syndrome treatment centers, village health teams (VHTs), caregivers (parents and guardians) of children with nodding syndrome and community members with no children affected with nodding syndrome in their families.

The district officials and healthcare professionals occupy special positions in the community and their opinions and knowledge about nodding syndrome including available treatment facilities and challenges encountered in planning and delivery of healthcare for nodding syndrome are crucial. Only district leaders who were members of the nodding syndrome task forces and directly involved in planning of response to nodding syndrome were included. Caregivers, and community members aged 18–34 and 35–50 years in Akwang and Atanga sub counties were identified by community gatekeepers including head of village health teams, local council leaders and health assistants based at the nodding syndrome treatment centers in Pajimo HC-III and Lacekocot HC-III in Akwang and Atanga sub counties respectively. The fifth category of participants was VHTs.

The parents and caregivers to the children and their immediate community members are in contact with the affected children and therefore are best placed to articulate the health seeking practices undertaken, sources of care used and the observed outcomes from the various sources of care. We included parents/guardians of children diagnosed based on the WHO diagnostic criteria for nodding syndrome [6] undergoing treatment at the established nodding syndrome treatment centers, and who had been in care for at least 3 months. Parents/guardians of children with nodding syndrome and nodding syndrome plus registered at the treatment centers were included. However, parents/guardians of children under care for less than 3 months were excluded because their experience of care was short and could still be adjusting to the diagnoses. We also excluded parents/guardians of children registered and receiving care at the centers but with diagnosis of epilepsy.

Regarding selection of village health teams for participation in focus groups; all VHTs in the respective study sites were eligible except those with self-reported/documented children with nodding children in their own families. We supposed that the “professional experiences” of such VHTs could perhaps be influenced by the presence of a child with nodding syndrome in the family. Technically the VHTs coordinate the communities with health facilities and therefore could have experiences of both worldviews.

### Data collection and procedures

Data were collected using focus group discussions (FGDs) and individual interviews. We carried out 24 in-depth interviews (IDIs) with caregivers, 7 IDIs with district leaders, 17 key informant interviews (KIIs) with healthcare professionals, and 16 FGDs with VHTs (4) and community members (12) (Table 1).

**Table 1:** Summary of study participants and methods of data collection

Participant category	Study district				Total
	Kitgum		Pader		
	Male	Female	Male	Female	
Caregivers (IDI)	7	6	6	5	24
Community members (FGD)	3	3	3	3	12
Village health teams (FGD)	1	1	1	1	4
Healthcare providers (KII)	4	5	4	4	17
District leaders (IDI)	3	0	4	0	7

We developed and used a study guide based on available literature on nodding syndrome. The guide was reviewed by experts in anthropology, epidemiology and public health to ensure the questions/contents are appropriate for the objectives and culture sensitive. The questions in the guide included aspects on: treatments usually given (at home, in the community, health units) to children with NS, duration of the various treatments enumerated, expected results from the treatments, and the challenges experienced by families with nodding syndrome because of the disease and during health seeking.

The same guide was used for all categories of participants. In-depth interviews and FGDs were conducted by four experienced research assistants with skills in data collection in social and health sciences researches. The four research assistants were trained for 2 days on nodding syndrome epidemiology and on this study objectives and procedures including data collection methods and consent procedures. The research assistants were university graduates with degrees in the field of humanities including social sciences, community psychology, and education; they were people who were born and spent part of their first 15 years of life in the study region, and were proficient in the local dialect (Acholi). This was to promote culturally sensitive approaches and gain detail information on participants’ perceptions and beliefs about nodding syndrome.

The study guide was double translated by two independent translators with expertise in both English and Acholi language. A third translator then harmonized the two Acholi versions to form the final guide/tool used in data collection. The final translated tool was tested for conceptual equivalence and completeness in data collection with two parents of affected children and a healthcare professional in Kitgum hospital nodding syndrome treatment center and one VHT from Akwang Sub County, Kitgum district. The four recordings from the pilot interviews were transcribed and translated. The investigators performed manual analysis of the transcripts to determine emerging themes and then refined the study guide.

Research assistants worked in pairs and there were two sets of paired research assistants; in each pair, one guided the interview or discussions process while the other took field notes and operated a digital audio-recorder. Each set of research assistants performed a maximum of four interviews/two FGDs per day. This was to ensure quality and allow recall of particular instances in individual interviews well enough during transcriptions. Data were collected during November and December 2013.

The interviews with district leaders were carried out in their offices while IDIs with caregivers were carried out in homes of participants. Healthcare professionals were interviewed in quiet rooms/offices within the health facilities where they worked. FGDs were carried out in community meeting venues including public halls, schools and health facilities. Key informant and in-depth interviews lasted about 45–60 min while the focus group discussions took about 60–90 min. Interviews and discussions were audio-recorded. Venues for both interviews and FGDs were carefully selected to minimize interruptions and maximize privacy for participants. Non-participants were not allowed at these venues.

### Data analysis

Data collected were transcribed and translated verbatim within the same week of data collection. Content analysis technique was used in data analysis; ATLAS.ti version 6.1 was used to support data analysis. Analysis started with three investigators reading through three different transcripts each. They independently formulated codes based on predefined themes in the interview guide and issues emerging from the data. The team of three then met and discussed the codes and emerging themes from the data. A codebook was formed, presented to the bigger team of investigators, discussed and a final version of the codebook developed. One investigator then read through all the transcripts several times and then applied the codes to meaning units. Data segments were retrieved and further analysis done by first author. The team of investigators then met to share findings and agreed on interpretation of themes. Key measures considered during analyses included perceptions on treatment practices for nodding syndrome and perceived barriers and enabling factors to health seeking at biomedical facilities.

### Ethical considerations

Ethical approval was obtained from Makerere University College of Health Sciences School of Medicine, Research and Ethics Committee (SOMREC). The Ministry of Health sanctioned this study and provided introduction letters to the districts before investigators started field work. During data collection, written individual informed consents were sought from the participants before interviews. Verbal consents were obtained from the participants in the FGDs and for audio-recording from all participants. Confidentiality was observed by the investigators. Participants were provided modest transport refunds after interviews and/or discussions. Investigators plan to go back to the field to disseminate findings to the communities involved in this research and people in the region.

## Results

All participants approached accepted and participated in the study. The general characteristics of participants are summarized in Table 1. Among all categories of participants, there was report of medical syncretism involving concurrent and/or alternating use of traditional herbs, traditional rituals, prayers and western medicines for treatment of children with nodding syndrome. Reported barriers to health seeking were similar across categories of participants. The barriers included health system factors e.g. long distances to facilities and lack of medicines, and contextual and societal factors related to affordability of treatment and beliefs about treatment modalities that can be used to treat nodding syndrome.

Representative quotes have been included, followed in brackets by participant transcripts order in the Atlas.ti software.e.g. P2, 25; method of data collection e.g. FGD; participant category e.g. community; and study site e.g. Akwang. Sex only included for the FGDs but not for individual participants to minimize chances of recognition.

### Treatment practices and cure of nodding syndrome

Consensus view from all five categories of participants was that nodding syndrome never cures with available current treatments.

“The illness started in 2004… and up to 2013 the disease is still there. They are also giving the drugs (western medicines) but I have not yet seen anyone of them who have got cured”, (P2: FGD, Community, Females; Atanga).

Caregivers reported that the western medicines do not cure nodding syndrome for there cannot be cure for a disease whose name is even not known by healthcare professionals; medicines are made for diseases with known names.

“No I have not yet heard of any which got cured. The healthcare workers tell us that they still do not know the disease and also the cause is still not known so the healthcare workers are giving us the medicine to treat epilepsy to treat luc luc (nodding syndrome), so there is no major change that the children can get now until we establish what the disease is and what causes it then we can now get the proper treatment for the children”, (P32: IDI, Caregiver; Atanga).

“I do not think the disease can cure; the only cure for the disease is the spades (death); when they are buried then that is the end of the disease and we have seen so many children who have died here. So this drug that we are getting is reducing its severity a bit but it is not curing”, (P35: IDI, Caregiver; Akwang).

Traditional medicines and rituals were also used but did not lead to cure of nodding syndrome. Communities also engaged in prayers but there was equally no cure.

“There was a time when we did some traditional rituals here with the help of the district local government… we did that but it all came to nothing; then even prayers, we have prayers in the community where even all the children come but there has been no cure. For us we have put it that the only cure that we shall see in the children is when they are buried because if it is taking the medications then the children have taken enough; there is no child who has cured that we have seen”, (P33: IDI, Caregiver; Akwang).

In all the village health team focus groups, participants expressed there is still no cure for the illness. The VHTs work with health facility outreach teams to supply medicines to affected families as well as go through the villages to check on children’s status. Perhaps the views of VHTs reflect both their own observations and experiences, and those of the communities in which they live.

“For cure we do not think it can come soon because if it were there then we could have known already. So we just want to see those changes like in those ones who are tied; even me I have given up on those ones because those ones cannot even think of untying themselves, they defecate there, eat from there and sleep there. Do you really see any hope of that one getting cured?” (P14: FGD, VHTs, Females; Atanga).

The healthcare providers also reported similar accounts that the western medicines provided are for symptoms control rather than cure of the illness.

“In the real sense, the treatment we are giving now is for the symptoms, there is no treatment to cure the disease now. What we are giving to the children is to enable them to do some little work or get up and play with the other children and make the life of the children easy but not to cure the disease”, (P44: KII, Healthcare Provider; Atanga).

### Perceived improvements with western medicines

Whereas cure was not observed yet, a common view from most caregivers and focus groups of the VHT was that western medicines lead to some improvement in symptoms.

“So even when we say the child sometimes improves it is not that he improves completely, it is just because the falling down and the jerking will disappear for some time but the mental disorientation and the entire disease will still be there. It is only the falling that sometimes disappears for a while but then it comes back”, (P16: FGD, VHTs, Males; Atanga).

Most of healthcare providers, and all the district leaders held the view that western medicines help reduce symptoms of nodding syndrome, especially convulsions. While the district leaders encouraged use of western medicines for treatment of nodding syndrome, there was a great concern among them that the mental functioning of the children do not improve well enough to allow them independent lives including self-care and feeding.

“When they take these drugs (western medicines) the frequency of the attacks reduces. They improve mentally but they will not have sound mind like ours”, (P49: KII, District leader; Kitgum).

“It is of recent that the ministry of health started intervening by giving this sodium valproate and that is when they started improving but the supply of the drugs is not consistent. When they default, the disease comes back seriously and they become worse than before… the improvement is there in the way they behave but their mental improvement is very slow”, (P51: KII, District leader; Pader).

Some district leaders reported that a few of the children have improved on the western medicines and have consequently resumed schooling.

“Some of the children have improved and gone back to school and some of them have even sat exams and they do not have frequent attacks now”, (P57: KII, District leader; Pader).

However, participants in only a few of community FGDs thought western medicines could lead to improvement in the illness symptoms.

“We should continue to give the drugs that we are giving currently even though people are saying that the drugs cannot cure but it is at least helping to reduce the severity of the disease”, (P11: FGD, Community, Males; Akwang 2).

### No improvement with current western medicines

In the majority of community FGDs, participants did not think the western medicines cause any reduction in symptoms of nodding syndrome and they wondered why the government is providing the medicines to the children because they have never seen any evidence that such medicines are helping the children. Such popular community views have the potential to discourage health seeking in health facilities and thus undermine government and stakeholders’ efforts to combat nodding syndrome.

“I do not know why the government sent these medicines here; whether to cure the disease or whether to just take the drugs. The children have now taken the drugs for too long. Some of them have now taken it for ten years and they are still taking; why isn’t the government changing the drugs to something else that will cause change in the children”, (P 5: FGD, Community, Females; Akwang_2).

“For me I want us to speak the truth, the changes that people see are in the Olili (epilepsy) but in the real luc luc (nodding syndrome) there are no changes… for me I do not see any changes in the children suffering from luc luc”, (P 8: FGD, Community, Males; Atanga).

Similarly, a substantial minority of healthcare providers also reported that there is no enduring objective improvements noted in the children with nodding syndrome.

“To be honest the issue of treatment is not easy for me to answer because the children have been receiving treatment now for two years but there is no change that I can see as a healthcare worker and get satisfied because there is no one who has come to me and said that my child is now okay. The child may recover and stay for about three months when he is fine but when the attack comes again it comes so strongly that it leaves the child in a very bad state”, (P56: KII, Healthcare Provider; Atanga).

### Use of alternative treatments

While only a few of the caregivers reported taking recourse to the power and cure from diviners, the majority of community members who apparently lost hope in the effectiveness of western medicines on nodding syndrome much early in the epidemic advocated for the use of traditional rituals, traditional medicines and spiritual healing. Majority of these community members however admitted that nodding syndrome has eluded the herbs that they used with success for similarly convulsive illnesses including epilepsy. Choice of herbs for new illnesses e.g. nodding syndrome was therefore based on their previous effectiveness on known illnesses with similar presentations to the new illness.

“Those men… so they came and killed the sheep and put it in an ant hill and we thought that the thing (ritual) would now cure the way it had helped the people of Pawena (who had a some other equally strange illness) but there were no changes in the children”, (P 4: FGD, Community, Females; Akwang_1).

“Like Mzee said, for ‘two olili’ (epilepsy) that we knew in the past, there was treatment. We had local herbs to treat it especially when it has just started and it would cure but this one which has now come even if you give whatever medicine it will not cure; some people are giving the local herbs but it does not cure it”, (P 9: FGD, Community, Males; Atanga).

For one caregiver, the solution for the illness was to be found by giving the child to eat the brain of a donkey. This caregiver joined the army so that he could get access to a donkey and extract its brain.

“There are these things that people say that would be used for treating epilepsy like the brains of a baboon and the brains of a donkey. All those things I looked for them until I joined the army from Pajimo barracks to get the brain of a donkey… I got the head and then gave the brain to my daughter but still she did not get cured. I joined the army because of that, I wanted to get the brain of a donkey,” (P38: IDI, Caregiver; Akwang).

### Perceived barriers to biomedical care for nodding syndrome

Two broad categories of factors affecting health seeking were reported by participants: (1) health system related factors which included inadequate skills of healthcare providers and negative attitudes, long distances to health facilities, limited access to safe modes of transport, need for repeated visits to health facilities, shortage of medicines, congestion and long waiting times at health facilities, and (2) contextual and societal factors including lack of money and illness fatigue.

### Health system related challenges

#### Inadequate skills of healthcare providers and negative attitudes

Some healthcare providers reportedly do not wish to work on children with nodding syndrome and sometimes exhibited negative attitudes towards caregivers.

“The issue of healthcare workers being rude to the patients is there; sometimes you go there and may be because they are tired they will tell you enough of those insults, but because you are the mother of the child who is sick you just keep quiet and pray that they give you the drugs and you go back home”, (P22: IDI, Caregiver; Atanga).

In Kitgum, there was less report of negative attitudes of healthcare providers towards the caregivers unlike in Pader. The majority of caregivers and community members were satisfied with the care and didn’t feel discouraged seeking care.

“There is no discrimination; if you go there you will get the medicine like the other people who are sick and when the child is so badly off then the VHTs call them (healthcare professionals) from town and they come with the vehicle and they take the child there. Like my daughter some time ago she was really ill so I went to the VHT and we called them then they came and collected us from here and we went to town where I nursed her for about two months”, (P34: IDI, Caregiver; Akwang).

#### Long distances to the health facilities

Some communities live far away from the health facilities. People from Atanga for instance reported that they walk long distances to access Atanga Health Centre III. All the caregivers and VHTs in Atanga expressed the problem of long distances. The district leaders, healthcare providers as well as community members reiterated the problem of long distances to treatment center.

“The problem is mainly the distance to the health facility; if you look at us here the nearest health facility is Atanga health center III and that is over 20 km to our village of Acamogoma and you can imagine walking with the sick child all that distance to the health facility, it is not easy”, (P9: FGD, Community, Males; Atanga).

However some communities such as those from Akwang reported that they did not have problems with accessing healthcare services because the drugs were brought to the community.

“There is that big mango tree… that is where we normally meet every after two weeks; that is where the healthcare workers come and assess the children and give them the drugs, it is not like in the past where we had to go up to Pajimo health center. I am sure you have also seen the distance. At that time some of the parents were already tired and would sometimes send the children on their own to the facility and the children would reach there with injuries only and then on the way back if the child gets an attack he/she may fall there then forgets the medicine. Now we have the medicines from here so there is no problem”, (P30: IDI, Caregiver; Akwang).

The problem of distance has been minimized by providing outreach services to the community. Nonetheless, VHTs reported that outreaches are limited and some villages are not served.

“There is a lot of problems in accessing the health center because at the moment the health center only conducts outreach in Tumangu village. The rest of the communities do no not have that privilege so they have to come all the way here to the health facility and yet it is really far and yet the healthcare workers insist that they want to see the children so that they assess him/her and see how he is progressing with the treatment they are giving”, (P15: FGD, VHTs, Males; Akwang).

Majority of the healthcare workers from Pader district also reported a similar problem of long distances and need for more outreaches. In addition, the roads are sometimes impassable.

“One of the problems is distance to the health facilities and also to the outreach points where they are supposed to get the services. If I look at Angagura there are four outreach points but they are still very far from some villages, so the number of outreach points should be increased to cater for all the villages. Then also the bad roads because we have the van but we cannot access some of the villages because of the bad roads”, (P62: KII, Healthcare Provider; Atanga).

The district leaders viewed the outreaches as an innovative approach to improving health seeking, improving adherence and keeping continuity of care for the children whose parents would otherwise fail to reach the health units. But they also reported inadequacy of the existing outreach posts in serving the majority population affected with nodding syndrome.

“The only thing I can say is the distance. Yes because even with the treatment centers, we have some homes that are even far from those centers; even if we go for outreaches they are still some distances from those centers and in a situation where it is a rainy season they have problems coming to the drug distribution centers”, (P49: KII, District leader; Kitgum).

#### Lack of appropriate and safe transport means

A challenge to reaching the treatment centers involve the actual means of transport; the nature of the illness characterized by sudden onset of convulsions and degree of disabilities in the children make the common means of transport—bicycles and motorcycles transport difficult and risky.

“The majority of them lack transport even if they desire to seek western healthcare… say you have two convulsing children you cannot put them on a bicycle. If you have two convulsing children that means there should be two other people holding them plus you the rider which is not possible. So that affects their health seeking behavior but for those whose children are stable, their health seeking behavior is much better”, (P48: KII, District leader; Pader).

Although there are service vans provided by the ministry of health to help the very sick children with transport, the caregivers, VHTs and community members reported that sometimes the parents have to pay for fuel. This issue prominently featured in Atanga but not in Akwang.

“The biggest problem that we face is carrying them to the health facility because like most of them cannot even sit on the bicycle, they are too weak to sit there… and you cannot even carry them on your back. And then also the vehicle that is there at the health center in Atanga they say that you should first of all put fuel in the vehicle and you find that from Atanga to come here may be like 40,000/= (11.5USD) which no one in the community can really afford; so it is very difficult”, (P29: IDI, Caregiver; Atanga).

#### Lack of medicines at facilities

There were reports of frequent lack of medicines at health units. The caregivers sometimes walked long distances to the facilities but found no medicines.

“The problem is that sometimes even when you want to give the child the drugs as prescribed, you will go to the health facility and they tell you that the drugs are not there which creates a gap in the treatment of the children; so what I think is that the drugs should be made available at the facility all the time”, (P3: FGD, Community, Females; Atanga).

Non-availability of anticonvulsant medicines have been minimized by increasing supply and thus ensuring continuity of care. The way this is done by the healthcare professionals is by rationing out whatever medicines available to at least all the children. This however does not go well with the parents who would prefer to get medicines enough for 2 or more months so that they have time to do other things and not just keep coming to the health centers every other week as if there are no other commitments.

“Today they are getting the medications well; the medicine come regularly and on time but the only problem that I see is that the medicines they are giving is not enough because they only give for one month and yet it is very far to go to the health facility every month. I would think that they should give the drugs for at least two or three months so that we don’t have to go there all the time”, (P23: IDI, Caregiver; Atanga).

The healthcare workers on their part were concerned with the frequent stock outs of sodium valproate which in their experience is much better in seizure control compared to other anticonvulsants e.g. phenobarbitone.

“Sometimes, like currently some of the drugs that we give them is out of stock; like the sodium valproate which normally helps them a lot has run out of stock and this is happening on many occasions where the facility runs out of the drugs”, (P45: KII, Healthcare Provider; Akwang).

#### Congestions and long waiting times

Participants especially caregivers noted that they often wait for long at health facilities to get medicines for their children because there are always many people.

“Then the third problem is that sometimes there are so many people in the health facility so you find that when we come in the morning you will go back home in the evening; sometimes the children are so hungry and they start getting the attacks there in the line”, (P27: IDI, Caregiver; Akwang).

The district leaders also reported the problem of congestions and long waiting times at the outpatient clinics because of shortages of healthcare workers. Some parents reportedly get discouraged when they wait for too long and the children start crying with hunger and yet they have nothing to feed the children with at the outpatient or outreaches. Some caregivers end up going back home without the treatments.

“The other big issue is waiting for the medicines because the healthcare workers are few… sometimes when the caregivers go to the outpatient they take long for the healthcare workers to see them quickly and then also at that point the children may be hungry and even the caregivers may be feeling hungry”, (P58: KII, District leader; Kitgum).

On the other hand, majority of the healthcare workers reported that they provide quick services for the children with nodding syndrome so that they go back home and have their meals.

“When they come we look at them as a special case and we attend to them first; it is also very easy to recognize them because you see they are different, so we pick them from the line because there is a special book for registering them separately so we pick them and register them, then we look into their medical form and then give them the medicines”, (P61: KII, Healthcare Provider; Akwang).

### Contextual and societal challenges

#### Lack of money

Most caregivers are subsistence farmers and majority reported lack of money as a major challenge in accessing healthcare for their children. Inability to pay for transport was a major constraint. A caregiver from Akwang had this to say:

“There is also the problem of money. There is nothing that you can do to get money, so we have to walk on foot to go and get the medicines and the health center is over 10 km away, that is what we do if there is no bicycle”, (P34: IDI, Caregiver; Akwang).

#### Illness fatigue and neglect of children

Some caregivers were reported to sometimes neglect the health needs, feeding and personal hygiene of their children. These children therefore tend to suffer other hidden unintended torments and distresses directly related to their having nodding syndrome.

“Most of the parents have lost hope. They do not have hope in the children who are sick. If there is a child who still does not have the disease in that home much of the hope of the family will be rested on that healthy child. The one who is sick does not receive the same or equal care and hope like the healthy children… and the fact that the disease attacks the brains of the children, people now take it that they are of no use to them”, (P15: FGD, VHTs, Males; Akwang).

The community and caregivers seem to no more view the nodding syndrome affected children as capable of keeping up to the values expected of children in the community. Children are needed not only as current economic assets for their labor power, but also as future assets for old age security.

“The best result that I want to see after the child has been given treatment is when the child is walking in the compound… and then I will continue looking after him. I really want to see him get cured and then he lives to help me in the future; also the same way I have taken care of him”, (P34: IDI, Caregiver; Akwang).

Similarly, the children with nodding syndrome who already have lots of functional disability are regarded as liabilities rather than assets.

“Yes they are not productive actually. I could say they are a liability to the family instead of being an asset to the family. They don’t contribute anything to the family and yet the family is always spending on them. They cannot even sweep the compound, they don’t even wash the plates, they don’t do anything, they don’t even dig and yet when you look at their age they are supposed to do all these things. So the families see them as a liability”, (P51: KII, District leader; Pader).

Healthcare providers also reported that some parents have really given up and either do not come or they send the children by themselves or some other older children or neighbors.

“Most of the parents are actually now tired. They say that they are tired because in the beginning they were coming to us at the health facility to get the drugs because they thought maybe they would see some major changes and the disease would cure but it is now some years and the children are not getting cured and also considering the distance to the health facility so some of them have now given up and stopped coming to the health facility”, (P44: KII, Healthcare Provider; Atanga).

The district leaders also reported the same problem of fatigue and noted that this potentially interferes with appropriate health seeking and adherence. Parents reportedly make remarks of desperation because desired improvements in illness are not seen.

“Some of them say this is a burden to them and the burden goes endlessly. Actually you could even hear the mothers say ‘I wish this one dies and I rest’. Because we don’t know what we are treating, we are only treating the symptoms but not the causes”, (P48: KII, District leader; Pader).

## Discussions

Treatment practices for nodding syndrome included traditional medicines; traditional rituals presided over by elders; prayers led by religious leaders; and use of western medicines concurrently and or in tandem depending on observed responses to the initial treatments. This is similar to other studies in Uganda in which it was found that western medicines have been used in combination with traditional medicines/herbs and rituals in treatment of many conditions including tuberculosis, diabetes, and cancer [25–27]. Medical syncretism is a common practice in Uganda and Africa in general [28]. Program managers, clinicians and policymakers may wish to take keen considerations of such inherent characteristics of the Ugandan society with respect to use of traditional methods of treatment which they often say have worked from time immemorial and predates the coming of western medicines.

In spite of the various treatment modalities being used in management of nodding syndrome, participants in this study reported that cure of the disorder is a farfetched outcome. In particular, the community members and the caregivers in both study districts believed that the only cure for nodding syndrome is the “spade”, referring to death of affected children. This may be an indication of grave desperation in a population that has undergone a prolonged spell of violent conflicts, destructions of homes and cultural set up and social safety nets with high levels of post traumatic disorders [29–32].

With regards to use of western medicines, the community members and caregivers recounted various types of medicines by colors and sizes. Some of the western medicines reportedly reduced symptoms especially that of convulsions and head nodding. But they reported the medicines were unaffordable only until about 2012 when Uganda government started providing them free of cost to the children. Most VHTs, healthcare providers and district leaders concurred that the western medicines, particularly sodium valproate (caregivers referred to it by color and time during which it came into use), reduced symptoms of convulsions, head nodding and led to regain of functional abilities in the children including personal body care e.g. eating on their own. A limited number of affected children had reportedly improved and gone back to school. Incidentally, the reported good responses were not long lasting; with time, symptoms got more severe and became associated with physical and mental retardations and deformities of the chest and legs. It has been reported before that symptomatic responses of nodding syndrome to western medicines especially anticonvulsants such as carbamazepine and phenytoin are neither curative, nor enduring and symptoms often worsened even when on treatment [4, 7, 11]. However, a more recent study in Uganda showed that use of western medicines including sodium valproate and associated physiotherapy and rehabilitation measures lead to sustained symptomatic improvement in children with nodding syndrome [15]. Therefore, caregivers and community members need ongoing encouragement to adhere to available treatment while researches into etiology and curative treatments for nodding syndrome continue.

Caregivers of children with nodding syndrome go through a lot of challenges during health seeking. Across categories of participants in this study, several challenges concerning health seeking for biomedical treatment were reported. Similar challenges were recently reported in a study involving community members and healthcare professionals with regard to health seeking for cervical cancer in northern Uganda [27, 33]. These practical challenges need practical and local solutions both in the immediate and long term. In general, access to treatment for chronic illnesses in Uganda and many other sub Saharan African countries are a major problem that requires a systems’ approach and probably an overhaul of the healthcare systems that have remained in the mode for dealing with infectious diseases that heal within short time and patients need no more long term follow-up. For chronic illnesses such as nodding syndrome and epilepsy, the system does not only need appropriate specialists distributed equitably and reachable when needed but also a system of data collection and keeping and follow up of patients so that continuity of care is ensured and quality of care becomes acceptable and satisfactory to the patients and their families. Similar challenges have been documented in management of epilepsy in Africa where specialist care are scarce, patients live far away from treatment centers and can hardly transport themselves to the facilities. In addition, it was noted that poverty prevents patients from buying the appropriate treatment as well as do prescribed investigations. Medicines are often not available in government facilities where children living with epilepsy would get treatment for free and recourse to traditional healers either in preference to biomedicine or because of inaccessibility of the biomedical facilities were common [34–36].

Lack of money for transport and other medical requirements was found to hinder health seeking for children with nodding syndrome. The people in the study region experienced a civil conflict which lasted more than two decades and during which people lost their means of livelihood and thus find difficulty making ends meet [20]. Poverty undermines access to healthcare in all its dimensions of geographical accessibility, availability, financial accessibility and acceptability both between and within countries in Africa and other low- and middle-income countries (LMICs) of the world [37]. Income generating activities and projects directed to support the families affected with nodding syndrome may contribute to restoring esteem and increase capacity to afford treatment and food items for the children with nodding syndrome.

### Strengths and limitations

This is one of first studies conducted by a multidisciplinary team and which used triangulation of data collection methods and participants’ categories to gain deep insights into nodding syndrome treatment practices and challenges encountered during help-seeking for the disorder. Viewpoints from the different categories of participants provided corroborating evidence. Triangulation of data sources and methods of data collection increases on the validity and trustworthiness of the information gained [38]. Focus group discussions and individual interviews are best suited to elicit experiences and beliefs while qualitative content analysis allows investigators to gain in-depth understanding of the latent contents of conversations of informants and can therefore help to inductively develop themes that capture the main concerns and challenges that need to be addressed in order to improve care [39, 40].

A limitation to this study is perceived sensitivity of the subject matter especially on the part of the civil servants particularly the healthcare professionals who at times expressed reservations. One healthcare professional reported that nodding syndrome has been politicized so much so that they would wish to simply manage the children with whatever is provided to them rather than contribute ideas on how to improve the practice environment and plan care. However, assurances and reassurances of confidentiality over the period of interviews allowed the healthcare professionals to open up and discuss issues freely.

It is important to note that, transferability of our findings need be done cautiously because our findings may be a reflection of the distress that the study population has undergone in relation to the prolong period of civil conflict and destruction of the socio-cultural fabric that usually provided the social safety nets in events of difficulties or calamities such as the outbreak of nodding syndrome.

## Conclusion

Multiple treatment approaches have been adopted for nodding syndrome but the disorder has yet eluded cure and has caused lots of distress to caregivers and communities. While the western medicines are reported to reduce symptoms of nodding syndrome, the glass seems half full because the medicines do not lead to cure of the disorder. Regarding access to care and motivations for health seeking, the glass seems half empty as there are several barriers to care seeking. Interventions to improve health seeking for children with nodding syndrome need to target the identified challenges and involve the operational level healthcare professionals more in planning of services. While more outreaches for treatment of nodding syndrome could be instituted to increase access to the disperse and largely poor population, community dialogues and group psychotherapies might help restore hope to the affected communities and increase adherence to medications.

## Abbreviations

FGD: focus group discussion
IDI: in-depth interviews
IDP: internally displaced persons
KII: key informant interview
LMIC: low- and middle-income countries
VHT: village health team
WFP: World Food Programme

## References

[1] Lacey M (2003). Nodding disease: mystery of southern Sudan. Lancet Neurol.

[2] Winkler AS, Friedrich K, Konig R, Meindl M, Helbok R, Unterberger I, Gotwald T, Dharsee J, Velicheti S, Kidunda A (2008). The head nodding syndrome—clinical classification and possible causes. Epilepsia.

[3] Winkler AS, Wallner B, Friedrich K, Pfausler B, Unterberger I, Matuja W, Jilek-Aall L, Schmutzhard E (2014). A longitudinal study on nodding syndrome—a new African epilepsy disorder. Epilepsia.

[4] Idro R, Opoka RO, Aanyu HT, Kakooza-Mwesige A, Piloya-Were T, Namusoke H, Musoke SB, Nalugya J, Bangirana P, Mwaka AD. Nodding syndrome in Ugandan children—clinical features, brain imaging and complications: a case series. BMJ Open. 2013;3(5). doi:10.1136/bmjopen-2012-002540. 10.1136/bmjopen-2012-002540 PMC3646179 23645924

[5] Iyengar PJ, Wamala J, Ratto J, Blanton C, Malimbo M, Lukwago L, Becknell S, Downing R, Bunga S, Sejvar J (2014). Prevalence of nodding syndrome—Uganda, 2012–2013. MMWR Morb Wkly Rep.

[6] WHO. International Scientific Meeting on Nodding Syndrome Kampala, Uganda, 30 July–1 August 2012. Meeting Report. http://www.who.int/neglected_diseases/diseases/Nodding_syndrom_Kampala_Report_2012.pdf 2012. Accessed 27 July 2014.

[7] Sejvar JJ, Kakooza AM, Foltz JL, Makumbi I, Atai-Omoruto AD, Malimbo M, Ndyomugyenyi R, Alexander LN, Abang B, Downing RG (2013). Clinical, neurological, and electrophysiological features of nodding syndrome in Kitgum, Uganda: an observational case series. Lancet Neurol.

[8] Buchmann K. ‘You sit in fear’: understanding perceptions of nodding syndrome in post-conflict northern Uganda. Glob Health Action 2014;7. doi:10.3402/gha.v7.25069. 10.3402/gha.v7.25069 PMC4212077 25361725

[9] Buchmann K (2015). ‘These nodding people’: experiences of having a child with nodding syndrome in postconflict Northern Uganda. Epilepsy Behav.

[10] Van Bemmel K, Derluyn I, Stroeken K (2014). Nodding syndrome or disease? On the conceptualization of an illness-in-the-making. Ethn Health.

[11] Mitchell KB, Kornfeld J, Adiama J, Mugenyi A, Schmutzhard E, Ovuga E, Kamstra J, Winkler AS (2013). Nodding syndrome in northern Uganda: overview and community perspectives. Epilepsy Behav.

[12] Nyungura JL, Akim T, Lako A, Gordon A, Lejeng L, William G (2011). Investigation into the Nodding syndrome in Witto Payam, Western Equatoria State, 2010. South Sudan Med J.

[13] MOH: Annual Health Sector Performance Report. Financial Year 2011/2012. http://health.go.ug/docs/AHSPR_11_12.pdf 2012. Accessed on 26 May 2013.

[14] Idro R, Musubire KA, Byamah MB, Namusoke H, Muron J, Abbo C, Oriyabuzu R, Ssekyewa J, Okot C, Mwaka D (2013). Proposed guidelines for the management of nodding syndrome. Afr Health Sci.

[15] Idro R, Namusoke H, Abbo C, Mutamba BB, Kakooza-Mwesige A, Opoka RO, Musubire AK, Mwaka AD, Opar BT (2014). Patients with nodding syndrome in Uganda improve with symptomatic treatment: a cross-sectional study. BMJ Open.

[16] Mutamba B, Abbo C, Muron J, Idro R, Mwaka A (2014). Stereotypes on Nodding syndrome: responses of health workers in the affected region of northern Uganda. Afr Health Sci.

[17] Dagne T. Uganda: Current Conditions and the Crisis in North Uganda. http://www.fas.org/sgp/crs/row/RL33701.pdf 2011. Accessed 20 Jan 2013.

[18] Lane R (2007). Northern Uganda: looking for peace. Lancet.

[19] McElroy T, Muyinda H, Atim S, Spittal P, Backman C (2012). War, displacement and productive occupations in northern Uganda. J Occup Sci.

[20] Horn R (2009). Coping with displacement: problems and responses in camps for the internally displaced in Kitgum, northern Uganda1. Intervention.

[21] Joireman SF, Sawyer A, Wilhoit J (2012). A different way home: Resettlement patterns in Northern Uganda. Political Geogr.

[22] Preux PM, Tiemagni F, Fodzo L, Kandem P, Ngouafong P, Ndonko F, Macharia W, Dongmo L, Dumas M (2000). Antiepileptic Therapies in the Mifi Province in Cameroon. Epilepsia.

[23] Iyengar PJ, Wamala J, Ratto J, Blanton C, Malimbo M, Lukwago L, Becknell S, Downing R, Bunga S, Sejvar J (2014). Prevalence of Nodding Syndrome—Uganda, 2012–2013. MMWR Morb Mortal Wkly Rep.

[24] MOH. Annual Health Sector Performance Report, Financial Year 2009/2010. http://www.health.go.ug/docs/AHSPR09.pdf 2010. Accessed 26 May 2013.

[25] Buregyeya E, Kulane A, Colebunders R, Wajja A, Kiguli J, Mayanja H, Musoke P, Pariyo G, Mitchell EM (2011). Tuberculosis knowledge, attitudes and health-seeking behaviour in rural Uganda. Int J Tuberc Lung Dis Off J Int Union Against Tuberc Lung Dis.

[26] Rutebemberwa E, Lubega M, Katureebe SK, Oundo A, Kiweewa F, Mukanga D (2013). Use of traditional medicine for the treatment of diabetes in Eastern Uganda: a qualitative exploration of reasons for choice. BMC Int Health Human Rights.

[27] Mwaka A, Okello E, Orach C (2015). Barriers to biomedical care and use of traditional medicines for treatment of cervical cancer: an exploratory qualitative study in northern Uganda. Eur J Cancer Care.

[28] Hausmann Muela S, Muela Ribera J, Mushi AK, Tanner M (2002). Medical syncretism with reference to malaria in a Tanzanian community. Soc Sci Med.

[29] Roberts B, Ocaka KF, Browne J, Oyok T, Sondorp E (2008). Factors associated with post-traumatic stress disorder and depression amongst internally displaced persons in northern Uganda. BMC Psychiatry.

[30] Roberts B, Odong VN, Browne J, Ocaka KF, Geissler W, Sondorp E (2009). An exploration of social determinants of health amongst internally displaced persons in northern Uganda. Confl Health.

[31] Pfeiffer A, Elbert T (2011). PTSD, depression and anxiety among former abductees in Northern Uganda. Confl Health.

[32] Branch A (2009). Humanitarianism, violence, and the camp in Northern Uganda. Civil Wars.

[33] Mwaka AD, Wabinga HR, Mayanja-Kizza H (2013). Mind the gaps: a qualitative study of perceptions of healthcare professionals on challenges and proposed remedies for cervical cancer help-seeking in post conflict northern Uganda. BMC Fam Pract.

[34] Wilmshurst JM, Kakooza-MwesigeA, Newton CR. The challenges of managing children with epilepsy in Africa. Semin Pediatr Neurol. 2014;21(1):36–41. doi:10.1016/j.spen.2014.01.005. 10.1016/j.spen.2014.01.005 PMC5496661 24655403

[35] Prevett M (2013). Epilepsy in sub-Saharan Africa. Pract Neurol.

[36] Mbuba CK, Ngugi AK, Newton CR, Carter JA (2008). The epilepsy treatment gap in developing countries: a systematic review of the magnitude, causes, and intervention strategies. Epilepsia.

[37] Peters DH, Garg A, Bloom G, Walker DG, Brieger WR, Hafizur Rahman M (2008). Poverty and access to health care in developing countries. Ann N Y Acad Sci.

[38] Shenton AK (2004). Strategies for ensuring trustworthiness in qualitative research projects. Educ Inform.

[39] Malterud K (2001). Qualitative research: standards, challenges, and guidelines. Lancet.

[40] Graneheim UH, Lundman B (2004). Qualitative content analysis in nursing research: concepts, procedures and measures to achieve trustworthiness. Nurse Educ Today.

